# Supplementary material for: The value of D-dimer and platelet-lymphocyte ratio combined with CT signs for predicting intestinal ischemia in patients with bowel obstruction
Source: PLoS One. 2024 Jul 8;19(7):e0305163. doi: 10.1371/journal.pone.0305163 (PMC11230572; doi:10.1371/journal.pone.0305163)
Supplement: Attachment — Submitted filename: Response to Reviewers.docx [file pone.0305163.s002.docx]

Dear Reviewer:

We wish to thank you for the time and effort you have spent reviewing our paper. We are pleased to note that you have found our research work interesting and also pointed out some problems to help us improve the quality of our study. Our itemized response to your questions, comments and suggestions is as follows:

**Question-1:** The inclusion criteria for this study should be more clearly described. This study focuses on cases that resulted in surgery as a result of bowel obstruction, but all cases that did not result in surgery seem to have no evidence of ischemia. It should be discussed how this point should be interpreted.

**Answer-1:** Our study is mainly based on surgical patients as the research object. For patients who have not undergone surgical treatment. There are two possible outcomes for those patients:①successful conservative treatment; ②failed conservative treatment. Firstly, patients with successful conservative treatment have been cured and discharged from hospital and returned to normal life. Secondly, there are only two outcomes in patients with conservative treatment failure: surgical treatment and abandonment of treatment (non-cooperation with treatment). Patients who underwent surgical treatment were included in this study, and patients who did not cooperate with the treatment met the exclusion criteria and were not included in the study.

**Question-2:** In this study, authors simultaneously perform multivariate analysis of categorical and continuous variables. It is unclear how the cut off value for continuous variables was set.

**Answer-2:** When Youden index (sensitivity + specificity -1) is the largest, the cut-off value of continuous variables is the boundary value**.**

**Question-3:** Were all cases registered in this study emergency surgical cases, or were conservative cases with no improvement also included? If cases without improvement by conservative treatment were registered, authors should consider timing from onset of symptoms to surgery as a factor in ischemia.

**Answer-3:** Our study includes emergency surgical cases and cases without improvement by conservative treatment. In this study, pain as a representative symptom of bowel obstruction was included. The relationship between duration of pain and intestinal ischemia was studied. The results showed that there was no statistical significance between the two (P < 0.05). (The results are shown in Table I and Table 3)

Special thanks to you for your good comments.

Yours sincerely,

Yuan Zhou
