# Supplementary material for: The value of D-dimer and platelet-lymphocyte ratio combined with CT signs for predicting intestinal ischemia in patients with bowel obstruction
Source: PLoS One. 2024 Jul 8;19(7):e0305163. doi: 10.1371/journal.pone.0305163 (PMC11230572; doi:10.1371/journal.pone.0305163)
Supplement: Attachment — Submitted filename: Response to Reviewers (1).docx [file pone.0305163.s003.docx]

Dear Editor:

Thank you for reviewing our paper and making some very useful suggestions. We have made revisions based on your suggestions and hope that you will review our paper again. I have reviewed the reference list and found no retracted articles. Our response to your comments is as follows:

**Comments-1:** please reflect more in the Discussion part.

**Anwser-1:** I've added the specifics to the Discussion part. Thank you for your suggestions!

**Comments-2:** Please provide details of how you determined the Cut-Off in the method section of the text more clearly.

**Anwser-1:** I've provided the details in the Statistical analysis section of the text instead of the method section.

**Comments-3:**What Chinese sentences are sometimes included in the text? Please delete them if they are unnecessary.

**Anwser-1:** I have deleted all Chinese sentences. Please forgive my carelessness.

Special thanks to you for your good comments.

Yours sincerely,

Yuan Zhou.
